# Cognitive Enhancement: Unanswered Questions About Human Psychology and Social Behavior

**DOI:** 10.1007/s11948-021-00294-w

**Published:** 2021-03-23

**Authors:** Eric Racine, Sebastian Sattler, Wren Boehlen

**Affiliations:** 1 Pragmatic Health Ethics Research Unit, Institut de recherches cliniques de Montréal (IRCM), 110, avenue des Pins Ouest, Montréal, QC H2W 1R7 Canada; 2 Department of Medicine and Department of Social and Preventive Medicine, Université de Montréal, 7101, Av du Parc, Montréal, QC H3N 1X9 Canada; 3 Departments of Neurology and Neurosurgery, Experimental Medicine, and Biomedical Ethics Unit, McGill University, 3801, University Street, Montréal, QC H3A 1X1 Canada; 4 Department of Sociology, University of Cologne, Universitätsstrasse 24, 50931 Cologne, Germany

**Keywords:** Enhancement, Well-being, Human flourishing, Health, Ethics, Social psychology.

## Abstract

Stimulant drugs, transcranial magnetic stimulation, brain-computer interfaces, and even genetic modifications are all discussed as forms of potential cognitive enhancement. Cognitive enhancement can be conceived as a benefit-seeking strategy used by healthy individuals to enhance cognitive abilities such as learning, memory, attention, or vigilance. This phenomenon is hotly debated in the public, professional, and scientific literature. Many of the statements favoring cognitive enhancement (e.g., related to greater productivity and autonomy) or opposing it (e.g., related to health-risks and social expectations) rely on claims about human welfare and human flourishing. But with real-world evidence from the social and psychological sciences often missing to support (or invalidate) these claims, the debate about cognitive enhancement is stalled. In this paper, we describe a set of crucial debated questions about psychological and social aspects of cognitive enhancement (e.g., intrinsic motivation, well-being) and explain why they are of fundamental importance to address in the cognitive enhancement debate and in future research. We propose studies targeting social and psychological outcomes associated with cognitive enhancers (e.g., stigmatization, burnout, mental well-being, work motivation). We also voice a call for scientific evidence, inclusive of but not limited to biological health outcomes, to thoroughly assess the impact of enhancement. This evidence is needed to engage in empirically informed policymaking, as well as to promote the mental and physical health of users and non-users of enhancement.

## Introduction

Stimulant drugs, transcranial magnetic stimulation, brain-computer interfaces, and even genetic modifications are commonly discussed as forms of potential cognitive enhancement (Farahany et al., 2018; Greely et al., 2008). Cognitive enhancement (or cognitive neuroenhancement) is defined as “the use of medications or other brain treatments for improving normal healthy cognition” (Farah, 2015, p. 379). This phenomenon is hotly debated in the public, professional, and scientific literature. Professional societies like the American Academy of Neurology (Graf et al., 2013) and advisory governmental bodies like the Presidential Commission for the Study of Bioethical Issues (Presidential Commission for the Study of Bioethical Issues, 2015) have investigated it and offered recommendations for clinicians and scientists (Allen & Strand, 2015).

Some proponents of enhancement claim that, in light of globalization – which is viewed as increasing competition and pressure for greater performance – all mental and material resources, including performance-enhancing methods, should be used to foster the intellectual, technological, economic, and cultural wealth of nations (Beddington et al., 2008). Similarly, on an individual level, increased cognitive capacity might lead to higher financial income and well-being (Bavelier et al., 2019; Beddington et al., 2008; Bostrom & Sandberg, 2009). Following this line of argument, cognitive enhancement is not only envisioned as a fully acceptable way of increasing cognitive function but is also considered equivalent to other methods of improving cognitive function, such as private education or information technology, and should even be strongly promoted and subsidized, i.e., allotted large-scale funding, as for education (Bostrom & Sandberg, 2009; Greely et al., 2008). Some opponents of cognitive enhancement consider its use and subsequent achievements unauthentic and unfair, especially in competitive settings or when access to the technology is selective or restrained (Sandel, 2007). Several opponents also believe that cognitive enhancement constitutes a major departure from current common and accepted forms of self-improvement, such that they threaten our evolution as a species or jeopardize significant cultural practices (Fukuyama, 2002; Kass, 2003; Sandel, 2004).

Many of the statements opposing cognitive enhancement rely on claims about human welfare and human flourishing. But with real-world evidence from the social and psychological sciences often missing, the debate about cognitive enhancement is stalled. In this paper, we describe a set of crucial debated questions about some of the psychological and social aspects of cognitive enhancement (see columns 1 and 2 in Tables 1, 2, and 3) and explain why they are of fundamental importance to address in the debate about cognitive enhancement and in future research. We voice a call for scientific evidence revealing the impact of enhancement on social and psychological outcomes (e.g., well-being, work motivation), as well as biological health outcomes. Just as the efficacy and safety of candidate cognitive enhancers should be further investigated outside of the lab and with more standardized and systematic research designs (e.g., regarding dosage, outcome measures, and sample size) (Battleday & Brem, 2015; Farah, 2015; Schleim & Quednow, 2018), so should their outcomes on psychological and social functioning.

**Table 1:** Debated Issues about Cognitive Enhancement and Related Unaddressed Research Questions: Psychological Outcomes of Cognitive Enhancement.^a^

Question to address	Explanation	Methodological and conceptual challenges	Possible studies	Examples of reference and inspiring studies^b^
Significance of psychological and social outcomes of cognitive enhancement	Arguments in favor of cognitive enhancement tend to equate the social and psychological outcomes of different methods, while arguments against it often stress their fundamental differences	Assessing social and psychological outcomes of cognitive enhancement	Interview studies with (non-)users about the expected and experienced social aspects of enhancement use, including stigma, perceived self-confidence, burnout, etc.	See Allen and Harocopos (2016) for an example of a semi-structured interview study in which motivations and social aspects of substance use are found. This may inspire future interview studies about cognitive enhancement users’ experiences
		Finding ways to compare outcomes	Psychological and cognitive tests assessing outcomes in cognitive enhancement users versus controls, testing, for example, depressivity, mood, anxiety, self-understanding, etc.	See DiLorenzo et al. (1999) and Morgan et al. (2010) for examples of studies assessing psychological, neuropsychiatric, and cognitive outcomes of either exercise or ketamine use. Similar studies could be done using cognitive enhancement and cognitive enhancement-specific outcomes instead
		Acknowledging the influence of contextual aspects of situations on the comparability of outcomes	Neuropsychological tests assessing the persistence and stability (long-term results), specificity (for cognitive capacities), and variability (effects on other skills and changes over time) of certain methods of cognitive enhancement (e.g., tDCS)	See Karok et al. (2017) for a study using neuropsychological assessment methods to determine the outcomes of tDCS. This could be helpful in designing a study to evaluate the neuropsychological outcomes of tDCS use for cognitive enhancement
		Distinguishing subjective and objective effects and studying their interrelations	Prospective studies also outside the laboratory evaluating primary outcomes of cognitive enhancement use (e.g., concentration or nausea), as well as secondary, long-term effects (e.g., life satisfaction or income)	See Castriotta et al. (2009) for an example of a prospective, observational study of Modafinil and other interventions (though without evaluation of peripheral outcomes)

^a^Non-exhaustive, illustrative set of questions to be addressed in research, methodological and conceptual issues, as well as proposed studies

^b^The example studies referred to in this section may be on topics other than cognitive enhancement, and not every aspect or section of these example studies may be relevant to proposed studies; they are included in this column because the methods/methodologies they make use of could be applied to studies of cognitive enhancement

**Table 2:** Debated Issues about Cognitive Enhancement and Related Unaddressed Research Questions: Different Methods of Cognitive Enhancement.^a^

Question to address	Explanation	Methodological and conceptual challenges	Possible studies	Examples of reference and inspiring studies^b^
Equivalence of different methods of cognitive enhancement	Arguments in favor of cognitive enhancement tend to deny the importance of enhancement methods while arguments against them often stress how they are inherently problematic	Integrating the importance of enhancement methods in ethical analyses	Prospective studies outside the laboratory evaluating and comparing the primary and secondary effects (i.e., side effects) of the use of different forms of cognitive enhancement, including technological methods (e.g., tDCS, neuropharmaceutical), conventional methods (e.g., mnemonic methods, coffee)	See Lavault et al. (2011) for an example of an observational study about effectiveness and side effects of Modafinil, which could be useful in designing a study to address the (side-) effects of Modafinil and other substances for cognitive enhancement use
		Finding ways to compare the methods	Surveys for the public and/or specific groups (e.g., physicians, enhancement users) aimed at understanding attitudes towards the use or users of different forms of cognitive enhancement as well as associated legal norms across time (e.g., using classical survey instruments on expectations regarding different enhancers or asking participants to respond to short scenarios, i.e., vignettes involving uses/users of different forms of enhancement)	See Fitz et al. (2014) for an example of a study using vignettes to understand public views on cognitive enhancement and Sattler et al. (2013a) for a study in which students were surveyed about the moral acceptability of (different types) of cognitive enhancement use.
		Evaluating the enhancement methods with sensitivity to evolving social, economic, and cultural contexts over time	Neuroimaging studies comparing the neurobiological effects of the use of different methods (differences in task-related neural activation or structural and functional changes during enhancer use)	See Ghahremani et al. (2011) for an fMRI study in which brain activity was compared in methamphetamine-dependent participants and controls

^a^See table note ^a^ in Table 1

^b^See table note ^b^ in Table 1

**Table 3:** Debated Issues about Cognitive Enhancement and Related Unaddressed Research Questions: Agent Motivations.^a^

Question to address	Explanation	Methodological and conceptual challenges	Possible studies	Examples of reference and inspiring studies^b^
Agent (cognitive enhancement user) motivations	Arguments in favor of cognitive enhancement tend to dismiss concerns about the effects of enhancement on agents and agency, while opponents often predict radical and transformative worrisome changes of moral agents	Recognizing the need to investigate effects of enhancement technology on agents and agents’ motivation without prejudging of its importance	Interview studies about cognitive enhancement uses and user motivations	See Allen and Harocopos (2016) for an example of a semi-structured interview study in which motivations and social aspects of substance use are found. This may inspire future interview studies about cognitive enhancement users’ motivations
		Integrating the effects of cognitive enhancement on well-being into ethical analyses	Surveys assessing changes in intrinsic and extrinsic motivation before and after (shortly and in the long run) enhancement use	See Rockafellow and Saules (2006) for a study examining the role of extrinsic versus intrinsic motivation (to participate in college athletics) in substance use
		Assessing the effects of cognitive enhancement on intrinsic and extrinsic motivation	Neuroimaging experiments characterizing activation patterns in intrinsically motivated behaviors versus behaviors motivated by cognitive enhancement user	See Lee et al. (2012) for an fMRI study comparing neural differences between instances of intrinsic and extrinsic motivation, which could guide future studies exploring this paradigm in the context of cognitive enhancement use
			Surveys for the public and/or specific groups (e.g., physicians, enhancement users) aimed at understanding the perceived and actual motivations and intentions of enhancement users as well as the evaluation/justification thereof (e.g., using classical survey instruments asking about stress, socio-economic conditions, culturally formed frames, goals and other potential motivators or asking participants to respond to short scenarios, i.e. vignettes in which individuals use cognitive enhancement for different reasons)	See Scheske and Schnall (2012) for a study in which university students judged the use of cognitive enhancers in healthy people and (Sattler et al. 2013a) for a study in which students were surveyed about conditions motivating the use and acceptance of cognitive enhancement.

^a^ See table note ^a^ in Table 1

^b^ See table note ^b^ in Table 1

### What are the Neglected Social and Psychological Issues of Cognitive Enhancement, Why Should we Address Them, and How?

In this paper, we use the ADC (Agent-Deed-Consequence) model of moral judgment as a guiding heuristic to help cluster issues associated with cognitive enhancement and structure current research and debates about it. According to the ADC model of moral judgment (Dubljević & Racine, 2014; Dubljević et al., 2018), human beings rely on intuitions about three inter-related components – agent, deeds, and consequences – when making moral judgments (i.e., assigning a label of “right” or “wrong” to a given situation).

In the case of cognitive enhancement, relevant “agents” are the (potential) (non-)users of the technology; they have agency as people, as well as individual traits, motivations, and intentions. Agents are judged as having a particular character (e.g., virtuous or unvirtuous). The term “deeds” would refer here to the (non-)use of cognitive enhancement and/or the specific methods of cognitive enhancement used; both can be judged as either “right” (more likely to be promoted and encouraged in society) or “wrong” (more likely to be discouraged or frowned upon). The term “consequences,” in this case, refers to the various potential individual and social outcomes of cognitive enhancement use. These can be judged either as “good” or “bad,” or as having positive or negative effects on the agent or others (Dubljević & Racine, 2014).

For each component, we discuss relevant issues of cognitive enhancement and the need for evidence-based research. Without careful and objective consideration of each of these, an integrative moral assessment and judgment about the use of cognitive enhancement cannot be made. We start with a discussion of consequences, as they seem to dominate the debate, followed by deed, then agent. It should also be noted that, though each of the three components are distinguished, they cannot be completely isolated from each other. In the end, it is an agent that performs a deed with certain consequences, and this is reflected in our discussion. We do not aim to provide a systematic review of the literature on cognitive enhancement, but to offer background and justification as to why certain important questions in the debate about cognitive enhancement should be answered and what kind of studies could be conducted to shed light on them.

### Consequences: Real Psychological and Social Outcomes of Cognitive Enhancement

#### The Problem and the Data Gap

The term “cognitive enhancement” implies that some products and devices are actually able to enhance cognition. However, the real-world psychological and social outcomes of enhancement technology are poorly documented and equivalently poorly understood from a basic science standpoint (Brühl & Sahakian, 2016; d'Angelo et al., 2017; Dresler et al., 2019; Farah, 2015; Grewal et al., 2020; Hopkins & Fiser, 2017;). In fact, even the basic efficacy of certain popular cognitive enhancers in healthy users is currently not well supported in the literature (Dubljević & Ryan, 2015; Kredlow et al., 2019). The discovered enhancement effects of most types of cognitive enhancement (pharmaceuticals, brain stimulation, etc.) vary strongly according to active ingredient or specific technology used, tested cognitive function, personal factors (such as typical level of baseline performance), and several other factors (Dresler et al., 2019). For example, the effects of Methylphenidate seem to be limited to certain cognitive functions, such as memory, while Modafinil was shown to better enhance cognition in sleep-deprived individuals than in well-rested individuals (Kredlow et al., 2019; Repantis et al., 2010). Moreover, and importantly here, what basic research reveals about human performance in experimental and controlled settings may not hold in real-world settings, as there are neither specific criteria established for determining ecological validity nor methods for ensuring that complex, real-life factors are considered. Take the example of memory improvement via pharmaceuticals versus memory improvement via mnemonic methods. Research might show (hypothetically) that cognitive enhancement pills and mnemonic methods are able to improve memory retrieval to a similar or identical degree. But it has been shown that the pills can negatively affect mood (i.e., self-rated contentment) (Marchant et al., 2009). This suggests the importance of developing finer-grain assessments of outcomes beyond those of primary interest, like memory or concentration. To our knowledge, limited evidence exists about such broader outcomes, and current outcome measures have, so far, been narrowly focused on cognitive functions with little attention to real-world contexts (Farah, 2015) and associated social and psychological “side effects” (Brühl & Sahakian, 2016; d’Angelo et al., 2017).

From a more holistic social and psychological standpoint, the outcomes of each intervention expand far beyond the single cognitive function tested to include individual and societal short- and long-term outcomes (e.g., changes in self-confidence and self-efficacy, stigma toward users, raised societal expectations for human memory and performance, indirect coercion to use drugs to not fall behind, or even burnout after long periods of high performance), that could be unintended or unanticipated (Vincent & Jane, 2018). For example, when cognitive enhancement pills are used in a real-life setting, rather than in a controlled research environment, the user may be stigmatized as “weak,” “stupid,” or “dangerous,” thus social interactions and relationships may be affected (Sattler & Singh, 2016; Bavelier et al., 2019), whereas it is rather unlikely that the real-life user of mnemonic methods will be stigmatized in this manner. Refusing the use of enhancement technologies in the case of, for example, a pilot on a difficult flight or of a surgeon doing a complicated surgery may result in other forms of stigmatization, such as blame for the outcomes (of the flight or the surgery) or coercion to use cognitive enhancement, as the availability of the technology may create expectations for its use, especially in high-stake situations (Sample et al., 2019). This negative consequence of the (non-)use of a distinct performance-enhancing method would constitute a measurable difference in outcomes and could be considered a “non-medical side effect” of this biotechnology.

### The Importance of the Gap in Ethical Debates

In ethical debates, some proponents of cognitive enhancement claim that the outcomes obtained via cognitive enhancement technology are equivalent to those obtained via other methods of improving cognitive performance (Greely et al., 2008). Accordingly, there would be nothing ethically special about the use of, for example, pharmaceuticals or brain-computer interface technology to augment one’s concentration or memory, in comparison to the use of the services of a tutor (Greely et al., 2008). Contrary to this view, some opponents of cognitive enhancement describe enhancement as a categorically immense departure from other methods of self-improvement, such that they represent radical threats to human nature, human culture, or both (Fukuyama, 2002; Sandel, 2004). But is the (non-)equivalence of outcomes established? Perhaps the outcomes are equivalent in terms of a measured ability to carry out a given task (e.g., memory function), but what about the effects on the broader functioning of the individual user or even unintended or unanticipated societal consequences? The honest answer is that we have limited evidence to directly support the views on either side of the debate. It is important to know if the consequences of cognitive enhancement on human behavior and social interactions differ in such a way that, for example, users of enhancement behave or are treated differently in these situations due to the potential effects (e.g., better memory or concentration), side effects (e.g., anxiety or euphoria), or social perceptions associated with different cognitive enhancement methods (Faber et al., 2017; Konrad-Bindl et al., 2016). It would also be paramount to know if cognitive enhancement use leads to outcomes (measurable by both objective and subjective methods) beyond cognition, such as financial reward, reduced or increased stress, and other predictors of well-being (Häusser et al., 2010; VanderWeele, 2017). When choosing between two different methods (e.g., a drug versus a tutor) to tackle a particular issue, medical side effects of the drugs should certainly not be ignored – and neither should the potential peripheral social and psychological outcomes of two different performance-enhancing methods.

### Proposed Studies to Fill the Knowledge Gap

Moving forward, we need more experimental studies that will assess not only the primary effects of cognitive enhancement use, but its social and psychological repercussions as well. Experimental psychology studies could comprehensively examine the effects of cognitive enhancers on different aspects of task performance, including whether improvement in one domain (e.g., rapid task switching) has collateral effects on other domains or abilities (e.g., switching between unpredictable tasks and tasks requiring sustained attention) (Marchant et al., 2009). There is much work to be done to move beyond general claims regarding enhancement of broad areas of cognition (e.g., memory, attention) to assess specific effects, their interactions, and their broader consequences. Also, as we know, experimental lab studies do not necessarily reliably predict real-world behaviors and, therefore, studies on the effects of cognitive enhancement should be conducted outside of the laboratory. Data should also be systematically collected in various settings (e.g., school, work) with different target tasks, group sizes, and group compositions, since the (non-)use of cognitive enhancers can affect and be affected by context-dependent factors such as work motivation, working norms, individual and group performance, stigma towards the self and others, and more, depending on situational and structural features (Faber et al., 2017; Ragan et al., 2013; Smith & Farah, 2011). Longitudinal observational studies with control groups could also be used to examine the effects of long-term cognitive enhancement use. See Table 1 for a (non-exhaustive) list of research questions, specific examples of potential studies that could be done, as well as corresponding methodological and substantial challenges.

### Challenges Moving Forward

Shedding light on real-world psychological and social outcomes of cognitive enhancers will not settle in itself ethical debates, unless ethics is narrowed down to the consideration of consequences irrespective of other dimensions involved. However, better data could help to either substantiate concerns about the outcomes of cognitive enhancement technology or invalidate them. One consideration moving forward is that experimental designs in which researchers provide participants with enhancers require careful ethics evaluations as there are risks associated with providing participants with enhancement methods (such as pills) that have been developed and often only approved for treatment purposes. The known risk profiles already established for the use of certain substances for treatment purposes may not apply when cognitive enhancement is used. The possibility of, for example, increased cognitive enhancement use over time and the corresponding likelihood of addiction, for example, would need to be examined in the context of cognitive enhancement use specifically. Barriers to conducting meaningful and representative research on cognitive enhancement (Brown, 2019; Forlini et al., 2013) could include the under-representation of different minority groups based on documented challenges in other areas of research (Diaz, 2012; George et al., 2014) in addition to the possibility of misalignment of enhancement with different cultural values (Brown, 2019). There are also foreseeable difficulties recruiting healthy subjects, difficulties securing funding, and the difficulty of justifying research in pediatric contexts given current research ethics guidelines (d'Angelo et al., 2017; Forlini et al., 2013; Weyandt et al., 2018). Surveying populations of existing (non-)users does not entail the same risks, but the usefulness of such studies for testing causal relationships is restricted.

### Deeds: The Equivalence of Different Enhancement Methods

#### The Problem and the Data Gap

The kinds of actions we (choose to) carry out, given a goal and a socio-cultural context, and the specific decision-making processes involved are crucial yet complex components of human psychology and behavior. To achieve the goal of improved cognitive functioning, there exist several distinct methods ranging from taking stimulant drugs to genetic engineering, each with potentially different associated intentions and motivations (see discussion about the Agent in the next section) and other psychological, social, and situational factors. For example, studying for an exam with the help of a tutor involves interacting with another person, paying them for their services, and actively listening to them. Studying with the help of cognitive enhancers involves procuring the necessary pill(s) (legally or illegally), taking the pill(s), and so on. In the second case, the situation from which an improved exam score may arise is very different from the first on social, financial, legal, and procedural levels, such that the deeds differ in numerous ways even if the outcomes may be similar. Agent-related factors such as the motivation why someone would choose to take cognitive enhancement pills rather than hire a tutor or consequence-related factors such as the persistence or reversibility of the enhancement effects are factors that in a sense may change the nature and the evaluation of the deed itself because they change its meaning to the agent and have different implications. Additionally, there could be differences in the conditions of use, such as the difficulty and legality of obtaining an enhancer or whether the enhancer is invasive (such as a pill) or non-invasive (such as a brain stimulation headband) (Medaglia et al., 2019). Other differences include the diverging moral evaluations of the practice in question, the interpersonal implications of methods in terms of access (to either the tutor’s services and/or the pill(s)), social acceptability, perceived emotions during information intake, and sustainability of the learned material, as suggested by investigations about moral enhancement (Specker et al., 2017).

Consider the analogous problem of performance enhancement in sports. Intensive training is looked upon more favorably than Erythropoietin (EPO). These two methods are judged differently within the given context of competitive sports, where there are certain expectations and rules (Schermer, 2008) and where performance gain by anything short of physical and psychological training would likely be considered a form of cheating (Schelle et al., 2014). Here, performance enhancement is widely perceived as an unethical method of achieving the goal of better performance, as it creates an unfair advantage over others (unless everyone has access to enhancement), because others have to put in more time and effort. But even with equal access, EPO use violates some of the intrinsic values in the context of competitive sports (e.g., if runners use scooters while others run, the running competition might be meaningless). Proponents of cognitive enhancement may advance strategies to actively neutralize the ethical salience of the different methods of achieving a goal by, for example, suggesting that certain current legally controlled or prohibited cognitive enhancers (e.g., Adderall) should be legally available to everybody, or that performance enhancement becomes legal in sports. Enhancement as a method could then be considered equivalent to other methods (Greely et al., 2008; Kayser et al., 2005). But again, a comprehensive and rigorous analysis of this equivalence of methods should come in support of these arguments; though equal and legal access to cognitive enhancers may solve a justice-related issue, there would remain issues related to the differences in terms of the motivational, psychological, procedural, and other aspects of enhancement. Furthermore, there are interactions between the agent (user) and the deed (method) as well as practical socio-political questions of how to distribute enhancers safely and fairly (e.g., with drugs in the drinking water or via coffee-shop-like models) (Dubljević, 2013). By the same token, those claiming that enhancement is condemnable in itself need to provide strong arguments and evidence supporting the notion that such a deed is intrinsically immoral or that the aspects listed above do in fact differ significantly between methods and affect the user in a meaningful way.

#### The Importance of the Gap in Ethical Debates

In ethical debates, the equivalence of biotechnological and non-biotechnological methods for achieving a given outcome has raised considerable controversy (Cole-Turner, 1998; Focquaert & Schermer, 2015). Even though ethical debates repeatedly evoke this issue, there is a dearth of evidence concerning different aspects of this debate. For example, surveys and experiments aimed at uncovering attitudes towards cognitive enhancers often inquire about safety, coercion, and fairness (unfair access, cheating, etc.), but less often examine explicitly the equivalence of different methods of enhancement and more importantly, if methods should even matter (Fitz et al., 2014; Funk et al., 2016; Schelle et al., 2014). Some of the more recent research suggests that methods sometimes do matter and sometimes do not, and that understanding how and why they matter is challenging. For example, the acceptability of using different technologies (e.g., pharmacological versus invasive brain stimulation) for enhancement varies (Fitz et al., 2014; Haslam et al., 2020), but it is unclear exactly why they are appraised differently. Some studies indicate that characteristics of the enhancement methods (e.g., the level of risk associated with taking a drug) do often—but not always—impact willingness to use such methods (Sattler et al., 2013b, 2014). Likewise, though willingness to use a substance-based enhancer has been found not to change across different types of substances with similar characteristics (e.g., an over-the-counter drug, a drug that is only available in pharmacies, a prescription drug, or an illegal drug), moral acceptability of their use was shown to differ (over-the-counter drugs seen as more acceptable than prescription drugs or illegal drugs, for enhancement purposes) (Sattler et al., 2013a). Other research found that the attitudes of physicians to prescribing different cognitive enhancers towards those cognitive enhancers, varies (Franke et al., 2014); also, in other stakeholders, attributes of a substance (e.g., its safety, legality, familiarity, efficacy) affected its ethical acceptability (Franke et al., 2014; Mayor et al., 2019). These diverging findings point to the complexity of the issue.

Yet the debate remains sometimes Manichean (Buchanan, 2011). On one side of the debate, the importance of methods is discounted: only the goals and end-results—not the methods—matter (Greely et al., 2008; Savulescu, 2005). At the other end of the spectrum, some argue that the methods used have overwhelming importance and are condemnable in themselves (Sandel, 2004). This latter view is in line with the deontological ethics tradition in which certain acts have intrinsic value (e.g., telling the truth) and others are intrinsically condemnable (e.g., lying). This kind of thinking is also reflected in formal norms and laws (e.g., by defining which actions are allowed or forbidden) and in informal norms and conventions, which help societies and smaller groups of people self-coordinate and avoid dilemmatic situations and conflicts by providing clear evaluative guideposts. Nevertheless, there is limited comparative analysis available of the impact of different methods of performance enhancement on actual human behavioral, psychological, and social factors. A fair amount of research is based on projected intents, not actual behaviors (Sattler et al., 2013a; Fitz et al., 2014). From a sociological and psychological standpoint, methods of enhancement matter because they shape the psychological context in which outcomes emerge (Rauthmann et al., 2015). This does not mean that all novel methods of human performance enhancement negatively impact users on a psychological or social level; it simply means that the potential for these effects (situational, social, psychological, etc.) cannot be discounted and should be systematically considered when making decisions about how new performance enhancement technologies should be used.

#### Proposed Studies to Fill the Knowledge Gap

To advance understandings of these aspects of human cognitive enhancement, we need prospective studies comparing users of cognitive enhancement technology (e.g., transcranial magnetic stimulation) with others using more conventional methods of cognitive enhancement. Furthermore, we must investigate not only the direct enhancement effects but also the peripheral effects of different cognitive enhancement methods on participants’ self-related measures such as self-efficacy and self-esteem, as well as more socially oriented measures (e.g., motivation to work with differently enhanced individuals or group performance). This kind of evidence is crucial in getting to the crux of the worries expressed in ethical debates and explore interactions between the deed, agent (user), and consequences. Moreover, experimental surveys using vignettes could assess public and expert judgments of different enhancement methods and related existing and requested formal norms. Ecologically relevant studies or direct observation in clinical or real-world settings would be useful complements. See Table 2 for more discussion about strategies to examine the (non-)equivalence of methods.

#### Challenges Moving Forward

Understanding the different impacts of methods of enhancement is difficult, because methods do not stand separately from their context of use and the intents and knowledge (about the methods themselves, the consequences of their use, etc.) of agents (users). Simplistic ethical analysis often presumes that certain deeds are inherently “good” or “bad,” but real-world morality is much more complex and calls into question the possibility of separating the intents behind a deed and the nature of a deed from its actual consequences (Dewey, 1984). Likewise, simply claiming an equivalence or non-equivalence between methods of enhancement forecloses genuine investigation into and discussion about the real impact of enhancement technology on human social and psychological practices, acknowledging here that deeds might be hard to separate from their implications. Too often, technology is approved or rejected based on its alleged intrinsic worth without much analysis of potential opportunity costs or of its broader impact on other existing practices and technologies (Buchanan, 2008). Take for example the historical opposition to all kinds of technological innovation (e.g., printing, electricity, sound recording). This opposition was often fueled by the fear of a loss (Juma, 2016). The flipside is that intrinsic value is sometimes attributed to all kinds of new technologies (e.g., stem cells, genomics, or artificial intelligence), based solely on hype and the belief in technological fixes (Caulfield, 2004a, 2004b; Caulfield & Condit, 2012; Nelkin, 1995; Priest & Talbert, 1994). We have now come to realize how innovations can reconfigure, often positively, the horizon of possibilities and opportunities for human beings (Jasanoff, 2004; Jasanoff & Kim, 2015) but we need to strike better equilibria between fear of innovation and hype toward innovation. Both attitudes are not ideal and need to be cross-checked by scientific evidence. As these examples demonstrate, the evaluation of the equivalence of methods can change over time and across situations, with changing values, social attitudes, and evolving understandings of the methods. This makes re-evaluations in certain intervals useful. To investigate and understand the true importance of enhancement methods, though, one must first grant that methods of enhancement are an important component of behavior, then study them accordingly.

### Agents: Motivations of Cognitive Enhancement Users and Non-users

#### The Problem and the Data Gap

Agency and the motivations underlying human performance enhancement matter from an ethical standpoint. Consider the differences between a situation in which the intention of the user of a performance enhancement technology is to gain advantage over other students in a competitive university entry exam and a situation in which the same technology is used to achieve a heroic feat with noble and altruistic intent that might save the lives of many people (e.g., landing a plane safely or performing a surgery accurately). These distinct motivations would likely entail very different moral evaluations; academic and legal institutions, peers, and society in general will treat each situation differently, indicating that regardless of whether intent *should* matter when it comes to cognitive enhancement use, it *does* matter.

Furthermore, the effects of the use of cognitive enhancers on agent motivation are also crucial to understand. One robust finding is that intrinsically motivated actions—actions performed for their own sake and for the sake of self-satisfaction, i.e., motivated by the value of actions themselves rather than for external reward—yield the most to the individual in terms of contentment and well-being, while extrinsically motivated actions (actions performed to obtain specific rewards) yield the least (Ryan & Deci, 2000). Studies of well-being and happiness show that individuals who report being happy, or who are reported as being happy and accomplished individuals by others, are those who are moved by deep convictions about life and its meaning (Ryff, 2014; Zika & Chamberlain, 1992) and thus, tend to be more intrinsically motivated. It is possible that some altruistic intrinsically motivated behaviors rely on distinct neural pathways, indicting an important difference between actions performed in deeper connection with intrinsic values and extrinsically motivated actions (Moll et al., 2006). Perhaps someone using cognitive enhancement to achieve a high grade on a university entrance exam will mainly be able to satisfy their extrinsic motivations, whereas someone who is studying and preparing for the exam without enhancement will likely reap more self-satisfaction from the process in and of itself. The receipt of an external reward (e.g., a high grade) may also be seen as at least partially attributable to the cognitive enhancement, further lessening the opportunity for self-satisfaction and intrinsic motivation (a phenomenon known as motivation crowding-out effect) (Frey & Jegen, 2001).

Another important question to consider is whether cognitive enhancement actually increases the user’s motivation to perform boring tasks, rather than boost target cognitive functions directly (Sahakian et al., 2015). This argument (and like-minded ones) warrants investigation into whether this increased motivation would also ultimately augment well-being, as would more intrinsically motivated actions. An unintended consequence of pill-induced motivation on agency could be that intrinsic motivation to perform tasks generally declines and that pills become essential in motivating particular action, akin to a phenomenon of addiction. Individuals might face a trade-off between greater attainments of outcomes and their sense of agency or self-efficacy. This could, in the end, reduce their well-being and autonomy by involuntarily increasing the motivation to work harder to realize unmotivating tasks (Bavelier et al., 2019).

Currently, we do not have an in-depth understanding of the impact of enhancement technology on motivation and, more broadly, on agents (users). However, we know that users of cognitive enhancers (in the form of drugs) tend to be motivated by increases in performance at work and in educational settings (Sattler et al., 2013b; Novak et al., 2007), despite rather unclear benefits of these drugs. Furthermore, other studies have suggested that stress, lower-than-average performance, low intrinsic motivation, peer influence, procrastination, and other factors tend to increase the willingness for enhancer use (Sattler et al., 2013a, 2014; Baum et al., in press). Some users may also be self-medicating (e.g., for attention deficits or memory problems) with cognitive enhancers, consciously or not, which could be another potential motivation for their use. Motivations could be influenced by hyperbolic media discourse about cognitive enhancers (Partridge et al., 2011), which could potentially also lead to significant placebo effects. These findings speak to the centrality of human intents and motivations in debates about enhancement technology.

#### The Importance of the Gap in Ethical Debates

Unfortunately, a commonplace rhetorical strategy used in debates about enhancement is to minimize (and sometimes dismiss) the ethical importance of agency and agent motivations and, instead, bring attention to the consequences of actions. Some proponents of moral enhancement, for example, claim without regard for the methods of enhancement and their differences or their potential side effects on agency that enhancement is essentially necessary for the end-goal of human well-being (Savulescu, 2005). This strategy relies on a narrow understanding of what brings happiness and fulfilment to flourishing individuals (Yaden et al., 2018), such as economic wealth, not to say anything about the gaps in evidence about the relationship between flourishing and cognitive enhancement (Buchanan, 2012; Persson & Savulescu, 2013). However, other scholars take the opposite route and tend to boil down the ethics of enhancement only to its motivational aspects such that the outcomes do not seem to matter (Sandel, 2004). These rather extreme strategies cloud the important roles of agents (users), their motivations, and what these motivations reveal about the agent when moral situations are analyzed more holistically and in an integrative manner, with situational, psychological, social, and other factors in mind (Dubljević & Racine, 2014). Motivations and intentions have importance because the level of blame assigned to an agent often depends on the agents’ intentions. This idea is embedded in several legal traditions (including Anglo-American criminal law). For example, the doctrine of *mens rea* (guilty mind) is part of the evaluation of the criminal responsibility of someone who has committed a criminal act. Likewise, people are usually interested in what is revealed about the person and their dispositions through their acts, and this is well-reflected in ethics theories that focus on agent characteristics (MacIntyre, 1984). In this sense, cognitive enhancement is often viewed negatively because it suggests unvirtuous behavior (Sandel, 2004). Though the motivations of those using cognitive enhancement (virtuous or not) are not the entire story (Dubljević & Racine, 2014; Dubljević et al., 2018), they need to be studied for their relative importance, as it appears that they do in fact matter in at least some situations (e.g., legal). Public opinion, as well as ethical analysis, may come up with different justifications or condemnations regarding various motives, and this might guide reactions towards the user, demands regarding policies, and user behavior. Furthermore, motivations to enhance need to be examined more carefully in light of their actual contribution to flourishing. For example, some argue that, in the context of technology use, that acceptance of the limitations of human condition is also part of a flourishing life (Parens & Johnston, 2019).

#### Proposed Studies to Fill the Knowledge Gap

Further examination of the motivations of those using cognitive enhancement, as well as the effects of cognitive enhancement on human motivation, is crucial from the standpoint of human psychology, sociology, and behavioral science. Agent motivation and other factors should also be examined in the case of addiction, which may impair agency itself (both self-attributed agency and agency attributed by others), as well as the ability to form and act on motivations. Some of this research could be accomplished by studies in behavioral neuroscience focusing on the mechanisms and neural pathways of different types of motivation, so that concrete and consequential differences between forms of motivation and their potential consequences can be pinpointed. Psychological studies could assess whether the intuitive notion that self-satisfaction is valuable applies to the case of cognitive enhancement and whether individuals using cognitive enhancement get the same sense of self-satisfaction from accomplishments as those who do not use it. Other studies could further establish the importance of social factors, such as perceptions of moral situations given agent intentions. Potential effects of cognitive enhancement on human motivation need to be investigated to inform enlightened and rational policy decisions. Experimental studies have been used to assess, for example, the impact of incentives on intrinsic motivation in many different contexts; although research ethics need to be carefully considered, such studies could be conducted in the context of cognitive enhancement and its effect on task involvement and intrinsic, as well as extrinsic, motivation to work on target tasks. Additionally, large-scale longitudinal survey studies may offer opportunities to prospectively assess the impact of cognitive enhancers on those who have started or will start using them and the relationships between motivations for their use and the lived stress, social and economic pressures, culturally formed framings, and goals of potential users. Further, interview-based and narrative social science research could help generate a deeper understanding of the impact of cognitive enhancers on actual users in comparison to non-users, while taking into account different values, orientations, and cultural backgrounds (Groeneveld et al., 2006). See Table 3 for suggestions for research on agency and agent user motivations.

#### Challenges Moving Forward

Research has provided some understanding of the motivations an individual may have for using cognitive enhancers, while mechanisms behind personal and situational drivers and hurdles, as well as their interplay, are still far from being sufficiently understood (Sattler, 2020; Sattler et al., 2013b; Baum et al., in press; Robitaille & Collin, 2016; Zelli et al., 2015). For the testing of causal hypotheses, experimental and longitudinal research is the medium of choice. However, assessing such motivations in situ might be difficult. Moreover, we have a very incomplete understanding of the effects of cognitive enhancers on human motivations. Shedding light on these matters may either alleviate or bolster worries that cognitive enhancers will radically change the structure of human motivation and thwart ideals of human excellence and achievement. Thus, effects on human motivation should not be presumed or discounted; the possibility that cognitive enhancement may be found to, for example, have no significant effect on motivational (or other) factors, should not preclude the need to test out whether this is the case empirically.

## Conclusion

The growth of biotechnology and neuroscience yields numerous possibilities for the development of cognitive enhancement. So far, debates about these possibilities involve important claims about the psychological and social outcomes of enhancement (consequences), the importance of enhancement methods used to attain a particular goal (deeds), and the role of agency and agent (user) motivations. These claims often stand as assumptions because they have not been sufficiently investigated. Yet, they are extremely important according to the ADC model that we have used as a heuristic to describe the ethical dimensions of cognitive enhancement. We have argued for (1) the study of a broad range of social and psychological outcomes associated with cognitive enhancers (in addition to biological and health outcomes); (2) investigation into the importance of the specific enhancement methods used, as they may have different social and psychological implications; and (3) greater consideration of agency and the role of agent motivation and its relationship to user well-being. These three components should not be investigated in isolation; their mutual dependency must be fully considered if a more realistic and comprehensive picture is sought. Importantly, regardless of study design, rigorous and scientific analyses must be based on open-mindedness about aspects of and arguments about cognitive enhancement regarding outcomes, deeds, and agent motivations – not only those which support a certain view or those valued in a given research protocol. An analysis based only on a priori favorable or unfavorable opinions about these aspects can succumb to biases (e.g., self-confirmation biases) and partial analyses (e.g., search satisfaction biases). Extensive research into these aspects is imperative if we are to assess the ethics of the (non-)use of cognitive enhancers in an evidence-based and integrative manner and inform future policy making as well as technology development. Defendable and rational policies concerning cognitive enhancement need to rely on strong and objective evidence exposing all aspects of cognitive enhancement, including its biological, legal, social, and psychological aspects.
